# Residues 39-56 of Stem Cell Factor Protein Sequence Are Capable of Stimulating the Expansion of Cord Blood CD34^+^ Cells

**DOI:** 10.1371/journal.pone.0141485

**Published:** 2015-10-27

**Authors:** Bin Shen, Wenhong Jiang, Jie Fan, Wei Dai, Xinxin Ding, Yongping Jiang

**Affiliations:** 1 Biopharmaceutical R&D Center, Chinese Academy of Medical Sciences & Peking Union Medical College, Suzhou, China; 2 Biopharmagen Corp, Suzhou, China; 3 Department of Environmental Medicine, New York University Langone Medical Center, Tuxedo, New York, United States of America; 4 College of Nanoscale Science, SUNY Polytechnic Institute, Albany, New York, United States of America; French Blood Institute, FRANCE

## Abstract

**Background:**

Stem cell factor (SCF) can stimulate hematopoietic stem cell (HSC) expansion; however, the specific structural region(s) of SCF protein that are critical for this function are still unknown. A novel monoclonal antibody (named 23C8) against recombinant human SCF (rhSCF) was previously found to inhibit the ability of rhSCF to enhance HSC expansion, making it possible to identify the relevant active region to HSC.

**Methods:**

Eleven polypeptides were synthesized, which were designed to cover the full-length of rhSCF, with overlaps that are at least 3 amino acids long. ELISA was used to identify the polypeptide(s) that specifically react with the anti-SCF. The effects of the synthetic polypeptides on human HSC expansion, or on the ability of the full-length rhSCF to stimulate cell proliferation, were evaluated ex vivo. Total cell number was monitored using hemocytometer whereas CD34^+^ cell number was calculated based on the proportion determined via flow cytometry on day 6 of culture.

**Results:**

Of all polypeptides analyzed, only one, named P0, corresponding to the SCF protein sequence at residues 39–56, was recognized by 23C8 mAb during ELISA. P0 stimulated the expansion of CD34^+^ cells derived from human umbilical cord blood (UCB). Addition of P0 increased the numbers of total mononucleated cells and CD34^+^ cells, by ~2 fold on day 6. P0 also showed partial competition against full-length rhSCF in the ex vivo cell expansion assay.

**Conclusion:**

Residues 39–56 of rhSCF comprise a critical functional region for its ability to enhance expansion of human UCB CD34^+^ cells. The peptide P0 is a potential candidate for further development as a synthetic substitute for rhSCF in laboratory and clinical applications.

## Introduction

Umbilical cord blood (UCB) has become a very important source of hematopoietic stem cell (HSC) transplantation post myeloablative and non-myeloablative therapies [1–3). Because of UCB stem cells’ higher tolerance of human leukocyte antigen disparity, relative to bone marrow or peripheral blood stem cell grafts, they provide a significantly greater chance of finding a donor for clinical hematopoietic stem cell transplantation [4, 5). However, the limiting cell doses provided by a single CB unit have been associated with delayed hematopoietic recovery for both neutrophils and platelets. Delayed neutrophil engraftment has been associated with early transplantation-related mortality primarily from infection [6, 7). For this reason, it is necessary to develop a safe and high-efficiency ex vivo expansion strategy, so that adequate amounts of stem cells can be generated and used for clinical applications [8–10).

Stem cell factor (SCF) is a growth factor encoded by the Sl locus on mouse chromosome 10 [11–13) and has been mapped to human chromosome 12q22-12q24 [14, 15). The structural organization of the SCF gene has already been reviewed [16, 17). SCF has been shown to significantly increase the survival and expansion of HSCs ex vivo and contribute to the self-renewal and maintenance of HSCs in vivo. Previous studies indicated that SCF can effectively expand HSCs ex vivo when supplemented with other cytokines, including thrombopoietin (TPO), FLT-3 ligand (FL-3L), interleukin 3 (IL-3) and granulocyte colony stimulating factor (G-CSF) [18–22). HSCs express the CD34 marker on their surface and the CD34^+^ cells are capable of colony formation, proliferation and self-renewal [23).

In our previous studies [24), by using a prokaryotic expression system, we obtained recombinant human SCF (rhSCF), which is capable of causing significant increases in CD34^+^ cell expansion. We then selected a novel monoclonal antibody (mAb) against rhSCF, named 23C8, through the use of hybridoma technology. We discovered that the binding of 23C8 to rhSCF leads to inhibition of rhSCF’s biological activity to expand CD34^+^ cells.

The specific epitope recognized by 23C8 may constitute a crucial region for the binding of rhSCF to and/or activation of its receptor KIT. Thus, a short polypeptide encompassing that epitope might have bioactivities similar to those of rhSCF. Experimental confirmation of this hypothesis is important, as the identified peptide could serve as the prototype for developing synthetic substitutes that are not only more economical, but also more potent, than the full-length rhSCF, for research and clinical applications.

In the present study, we synthesized overlapping polypeptides of rhSCF and employed ELISA to identify the specific polypeptide(s) that positively react with 23C8. In addition, each polypeptide was also tested for its ability to stimulate the expansion of human UCB-derived CD34^+^ cell ex vivo. We found that the polypeptide corresponding to amino acid 39–56 of rhSCF (named P0) was the only peptide that interacted with 23C8 in ELISA, or enhanced human UCB CD34^+^ cell expansion ex vivo. Our results indicated that amino acid 39–56 is a critical functional region for enhancing human UCB CD34^+^ cell proliferation. Our findings suggest that the peptide P0 is a good prototype for the development of synthetic substitutes for rhSCF.

## Materials and Methods

### Ethics Statement

All research involving animals was conducted according to relevant national and international guidelines. Female BALB/c mice (specific pathogen-free; 8–10 weeks old, weight 18.0 ~ 25.6 g), obtained from the Experimental Animal Center of Soochow University (Suzhou, China), were used for mAb production. The experiment protocols were approved by the Institutional Animal Care and Use Committees of Soochow University (IACUC permit number: SYXK(Su) 2012–0045), and were in accordance with the Guidelines for the Care and Use of Laboratory Animals (National Research Council, People’s Republic of China, 2010). We further attest that all efforts were made to ensure minimal suffering.

All fresh UCB samples by written consultation of volunteer patients were provided by Suzhou Municipal Hospital (Suzhou, China). The consultation forms were signed by the participated patients. The study and all necessary signed forms were approved by the Hospital's Ethics Committee and Research Ethics Advisory Committee.

### Cell lines and reagents

The PQE60 plasmid and *E*.*Coli* BL 21 were obtained from QIAGEN (Valencia, CA, USA). Isopropyl-1-thio-ß- D-galactopyranoside (IPTG) was from Bio-Basic Inc (Amherst, NY, USA) to induce recombinant SCF expression in bacteria. Guanidine Hydrochloride was from Amresco Inc (Solon, OH, USA). Urea was from Shanghai Experimental Reagent Inc (Shanghai, China). RPMI 1640 medium and STEM PRO®-34 SFM medium were from Gibco (Grand Island, NY, USA). Fetal bovine serum was from Hyclone (Logan, UT, USA). Goat-anti-mouse immunoglobulin G (IgG) conjugated with alkaline phosphatase was from Biolegend (Canada). Prestained protein molecular weight marker was from Bio-Rad (USA). Biotin conjugated goat anti-mouse-IgG was from Biolegend (San Diego, CA, USA). Mouse monoclonal antibody isotyping reagent kit and streptavidin-peroxidase was from Sigma-Aldrich (St. Louis, MO, USA). MACS immunomagnetic absorption column separation device and CD34 MicroBead Kit, was from Miltenyi Biotec (Germany). rhTPO and rhFLT-3 were from Pepro Tech (USA). Anti-human CD34 mAb conjugated with phycoerythrin was from Immunotec (Canada). Peptides were synthesized by Nanjing Genscript Inc. (Nanjing, China).

### UCB collection and CD34^+^ cells isolation

Fresh UCB samples were obtained within 6–8 hours of delivery from Suzhou Municipal Hospital (Suzhou, China). UCB CD34^+^ cells were isolated from total mononucleated cells (MNC) with the MACS immunomagnetic absorption column separation device and CD34 MicroBead Kit, according to the manufacturer’s instructions (Miltenyi Biotec, Germany). MNCs were obtained by density centrifugation, with use of Ficoll-Hypaque Premium reagent (GE healthcare, USA). The purity of CD34^+^ cells was verified using flow cytometry, with an anti-human CD34 mAb conjugated with phycoerythrin (PE, Immunotec, Canada) and a model BD FACSVerse flow cytometer (BD, USA).

### rhSCF and mAb 23C8 generation

Prokaryotic expression system was utilized to produce rhSCF as described previously [21, 24). Briefly, BL21 *E*. *coli* transfected with rhSCF-expressing vector was proliferated in 2 x YT medium (250 rpm shaking, 37 ^o^C). rhSCF expression was induced by isopropylthio-b-d-galactoside (IPTG) at final concentration of 1mM. Purified rhSCF was obtained after dialysis of inclusion body against a serial of refolding buffers, CM Ion Exchange Chromatography, and Supersex-75 chromatography [25). rhSCF with biological activity was used for mAb production in BALB/c mice, using standard methods developed in our laboratory [26). Spleen cells from immunized mice were fused with sp2/0 myeloma; the hybridomas were cultured in hypoxanthine-aminopterin-thymidine (HAT) medium, and the supernatants of the culture were screened for affinity toward rhSCF using ELISA. Positive cultures were then limiting-diluted for isolation of mAb cell lines. The mAbs obtained from the cell culture supernatant of individual mAb cell line were further purified through Protein G affinity chromatography according to the standard IgG protein purification protocol. The obtained mAb 23C8 was identified via Western blots for its specificity. A mouse monoclonal antibody isotyping reagent kit (Sigma, USA) was used to identify the mAb subtype.

### Peptide synthesis and ELISA

Eleven polypeptides were designed and synthesized (Sangon, Shanghai and Genscript, Nanjing), with 3–9 amino acids overlap, to cover the full length of rhSCF. They were P0: 39–56; P1: 1–18; P2: 10–24; P3: 22–44; P4: 50–72; P5: 70–92; P6: 89–107; P7: 103–125; P8: 121–146; P9: 142–160; P10: 152–164. ELISA was performed using standard protocol as described previously [26). Briefly, 1 μg of each peptide, or BSA, or purified rhSCF protein, in 100 μl, was used to pre-coat wells of a 96-well-plate at 4°C overnight. The 23C8 mAb was applied at a 1:500 dilution, and incubated at 37°C for 1h. All tests were carried out in triplicates. Production of rhSCF in E.coli and purification using gel filtration, and production of mAb (23C8) and further purification using protein G affinity column, has also been described previously [21, 26).

### Test of ex vivo expansion of UCB CD34^+^ cells

Human CD34^+^ cells were isolated as described above, and then cultured (6.4 X 10^4^ cells in each well in a 24-well-plate) in 1 ml of STEM PRO®-34 SFM medium, supplemented with 10% fetal bovine serum, 100 ng/mL Pen/ Strep, and 2 mM L-glutamine (Gibco, USA), as well as 20 ng/ml rhTPO and 100 ng/ml rhFlt3-L (Pepro Tech, USA). Additional reagents were added where indicated, including rhSCF protein (100 ng/ml) and peptides (100 ng/ml, each). Regarding to mAb addition groups, 100 ng/ml rhSCF combined with 23C8 was added at 0.1, 0.2, 0.5, 1.0 and 2.0 molar ratio to rhSCF; 100 ng/ml P0 along with 23C8 was added at 0.1, 0.2, 0.5 and 2.0 molar ratio to P0. The mAb or the buffer was pre-incubated with rhSCF or P0 at 37°C for 0.5 h before they were added to the assay wells. All groups were cultured for 6 days in an incubator with humidified air containing 5% CO_2_. Culture medium was replenished on the third day post planting. Total cell number was monitored using hemocytometer, whereas CD34^+^ cell number was calculated based on the total cell number and the proportion of CD34^+^ cells determined via flow cytometry (BD, USA) on day 6 of culture, using the anti-human CD34 monoclonal antibody conjugated with PE fluorescein (Miltenyi Biotec, Germany).

### Colony Forming Unit Assays

MethoCult H4230 methylcellulose medium (Stem Cell Technologies, Vancouver, Canada) were thawed overnight in a 4°C refrigerator. Six-day-old cells from FT, FT+rhSCF and FT+P0 groups were prepared at 11X of the final concentration required. Then, 0.3 mL of the cells were added to 3 mL of MerhoCult medium; duplicate cultures were prepared using 1.1 mL of the cell suspension in each 35-mm dish. The cells were incubated for 14–18 days at 37°C under 5% CO2 and > 95% humidity. The various colony-forming units (CFU), including the erythroid burst-forming units (BFU-E), the granulocyte-erythrocyte-macrophage-megakaryocyte colony forming units (CFU-GEMM), and the granulocyte-monocyte colony forming units (CFU-GM), were observed with a bright-field microscope, 16 days after the cells were plated in MethoCult medium. A colony with >100 cells was counted as a positive colony.

### Competition assay

Human CD34^+^ cells were purified as described above, and then cultured (7.0 X 10^4^ cells in each well in a 24-well-plate, n = 4) as described above. There were two experimental arms: one with rhSCF (100 ng/ml) addition, the other without. P0 was added to both arms to final concentrations of 0–100 ng/ml.

### Statistical analysis

One-way analysis of variance (ANOVA), followed by Dunnett’s multiple comparison test, was used for comparisons among the various groups. Results are considered statistically significant when P value is less than 0.05.

## Results

### Production of rhSCF and specificity of anti-rhSCF mAb

**Fig 1:**
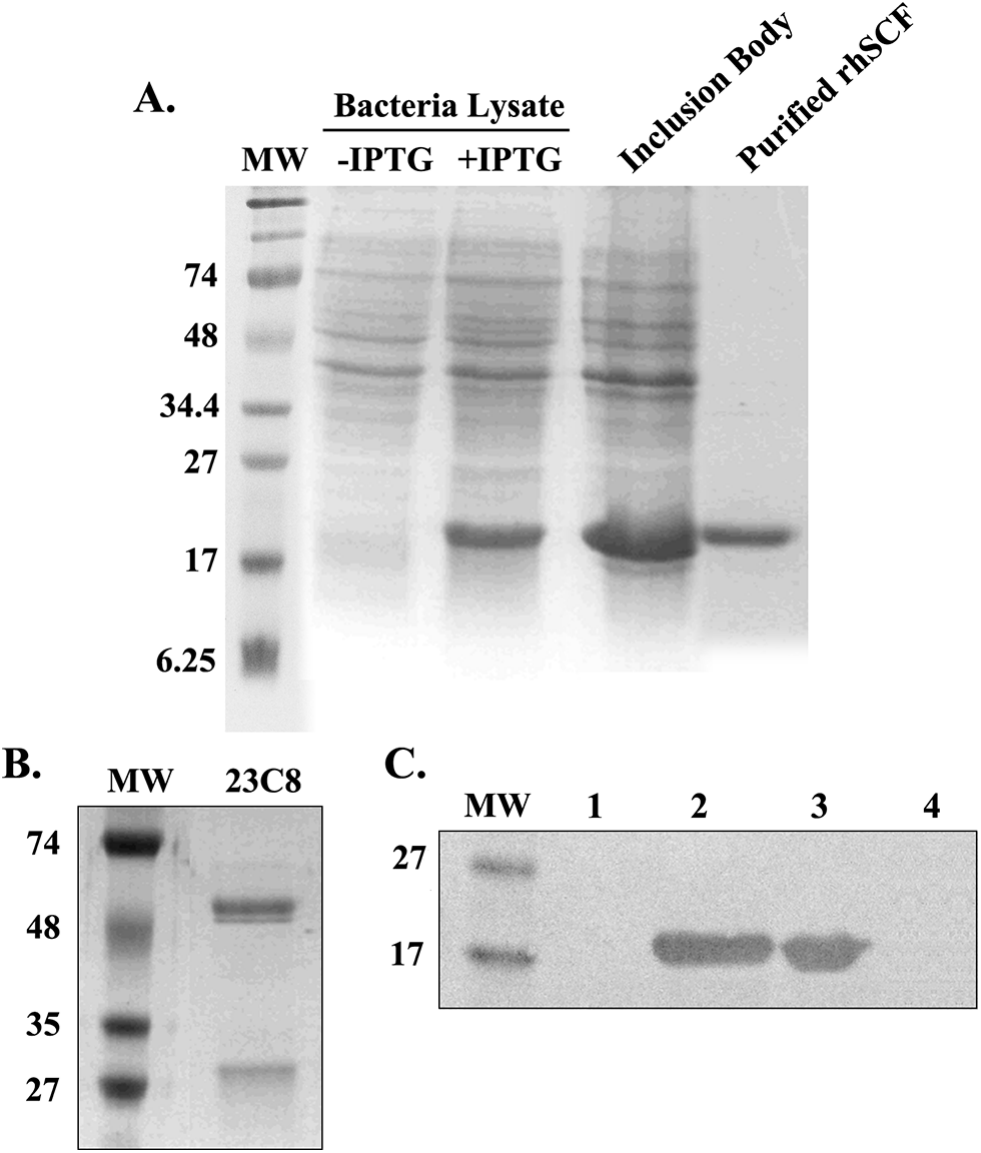
Purity of rhSCF and specificity of anti-rhSCF. (A) SDS-PAGE of purified rhSCF protein (Coomassie Blue staining). Lane 1: Prestained molecular weight markers Lane 2:Non-induced bacterial lysates. Lane 3:IPTG Induced bacterial lysates; Lane 4: Inclusion body; Lane 5: Purified rhSCF protein (5μg protein loaded). (B) SDS-PAGE of purified monoclonal antibody 23C8 (10 μg protein loaded; Coomassie Blue staining). (C) mAb 23C8 specifically recognized rhSCF, but not other protein, in Western blot analysis. Samples loaded in lanes 1 to 4 were Non-induced bacterial lysates, rhSCF Inclusion body, Purified rhSCF protein, and Purified G-CSF protein, respectively. Purified rhSCF and G-CSF were loaded at 10 ug for each lane.

rhSCF was obtained from inclusion bodies following IPTG-induced expression in *E*.*coli*. High purity of rhSCF protein was obtained after gel filtration of Supersex-75 chromatography (Fig 1A). A hybridoma cell line was selected to be positive for rhSCF in ELISA. Culture supernatant was collected and mAb 23C8 was further purified using protein G affinity column (Fig 1B). The specificity was also confirmed by western blot assay (Fig 1C). The anti-rhSCF mAbs did not detect any bands in total *E*. *coli* cell lysate (in the absence of IPTG), and did not cross-react with rhG-CSF either. The antibody detected one band with expected molecular weight for rhSCF (~ 18 kD) in rhSCF-expressing *E*. *coli* cell lysate (induced with IPTG) and inclusion bodies, as well as the purified rhSCF, indicating mAb 23C8 was the specific antibody against rhSCF and it was further subtyped as mouse IgG1 by ELISA analysis.

### Inhibitory effect of mAb 23C8 on rhSCF stimulating CD34^+^ cell ex vivo expansion

**Fig 2:**
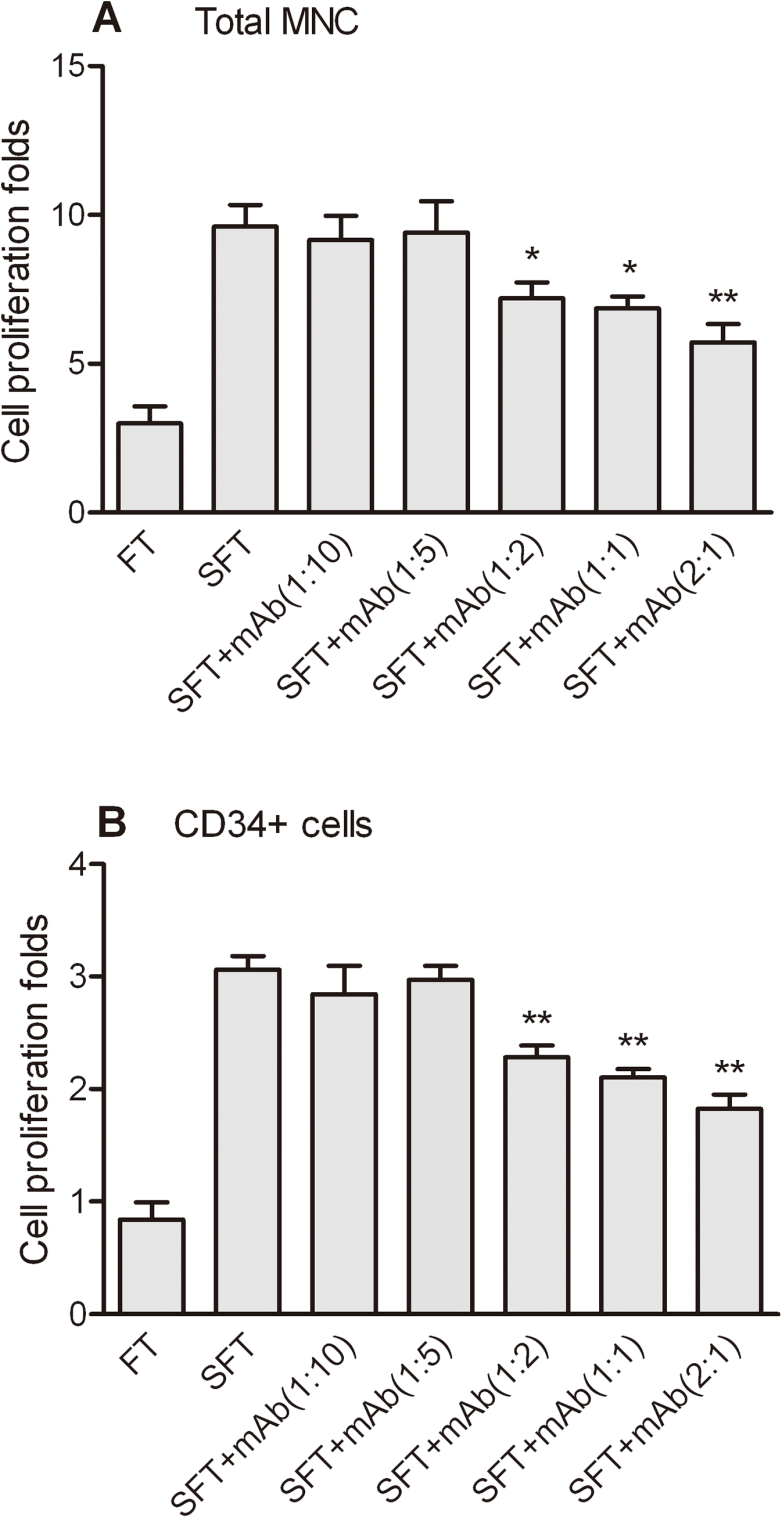
Inhibition of the activity of rhSCF by the monoclonal anti-rhSCF antibody. UCB CD34^+^ cells were affinity-purified and cultured with StemPro basal medium supplemented with Flt-3 ligand and TPO (FT). Media was supplemented with 100 ng/ml rhSCF where indicated (SFT). Anti-rhSCF mAb was added at 0.1, 0.2, 0.5, 1 and 2 molar fold to rhSCF. Total nucleated cell (A) and CD34^+^ cell (B) proliferation folds were determined on day 6. Cell proliferation fold was calculated as the fold increase [(after expansion)/(before expansion)] in cell counts. One-way ANOVA followed by Dunnett’s Multiple comparison test. Data represent means ± SD, n = 4 *, **, P<0.05, P<0.01, respectively, mAb groups compared to SFT group.

rhSCF significantly increased the expansion of total cells and CD34^+^ cells at final concentration of 100ng/ml supplemented with other two cytokines, Flt3-L, and TPO [24). The rhSCF enhanced the total cells expansion folds [from 3.00±0.81 to 9.60±1.04 (P<0.05)], and CD34^+^ cells [from 0.84±0.21 to 3.06±0.17 (P<0.05)] on day 6. The activity of rhSCF can be neutralized by mAb 23C8 at molar ratio of more than 0.5 molar fold over rhSCF (Fig 2). This result indicates that the mAb 23C8 can competitively inhibit the enhancement of rhSCF to CD34^+^ cell expansion.

### Identification of peptide(s) that interact with the anti-SCF 23C8

**Fig 3:**
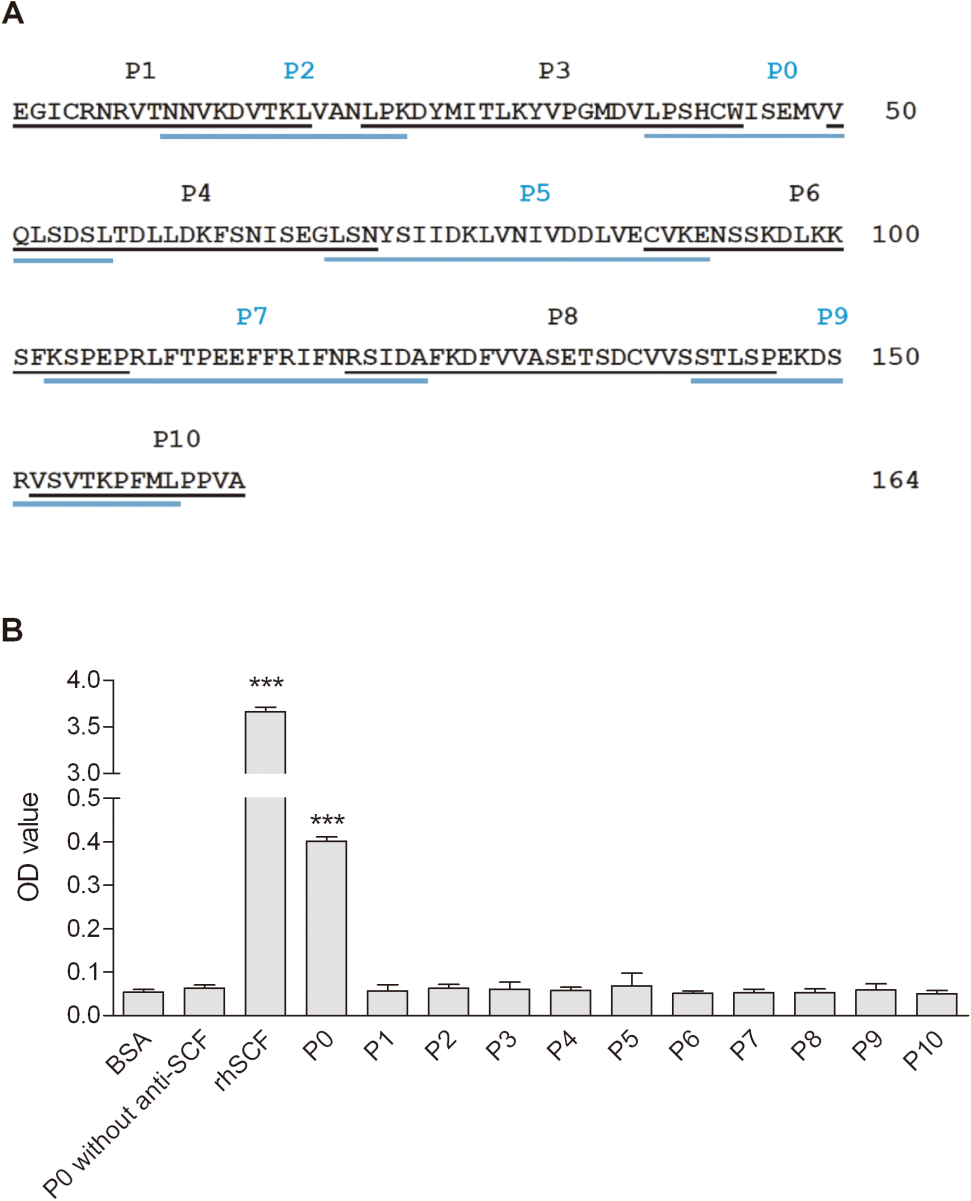
ELISA analysis of a panel of synthetic overlapping peptides covering the entire rhSCF sequence. (A) Position (underlined) of peptides along the rhSCF sequence. Eleven overlapping peptides (P0-P10; ranging from 13 to 26 amino acids) covering the full length of the soluble rhSCF protein were synthesized. Peptide 0 also contained three additional lysine residues (not shown; two at N-terminus and one at C-terminus) that were not present in SCF. Residue numbers are labeled to the right. (B) ELISA analysis of the synthesized peptides for binding with the monoclonal antibody 23C8. BSA and rhSCF proteins were used as negative or positive controls, respectively. Another negative control was coating with peptide P0, but omitting the mAb. Data represent means ± SD, n = 4. ***, P<0.001, compared to BSA control group. One-way ANOVA followed by Dunnett’s multiple comparison test.

A library of 11 polypeptides covering the full length of the rhSCF protein was synthesized. These were designed to overlap by a minimum of three amino acids, as shown in Fig 3A. For P0, which was difficult to purify due to inherent high hydrophobicity and low isoelectric point, two lysine residues were added to the N-terminus and one lysine was added to the C-terminus. All peptides were tested at the same concentration (100 ng/ml) using ELISA, with use of rhSCF as the positive control and bovine serum albumin (BSA) as a negative control (both added also at 100 ng/ml). An additional negative control was included in which P0 was tested without the addition of 23C8. As shown in Fig 3B, P0, of all peptides tested, was the only one that was detected by 23C8.

### Ability of the synthetic peptide(s) to stimulate UCB CD34^+^ cell expansion

**Fig 4:**
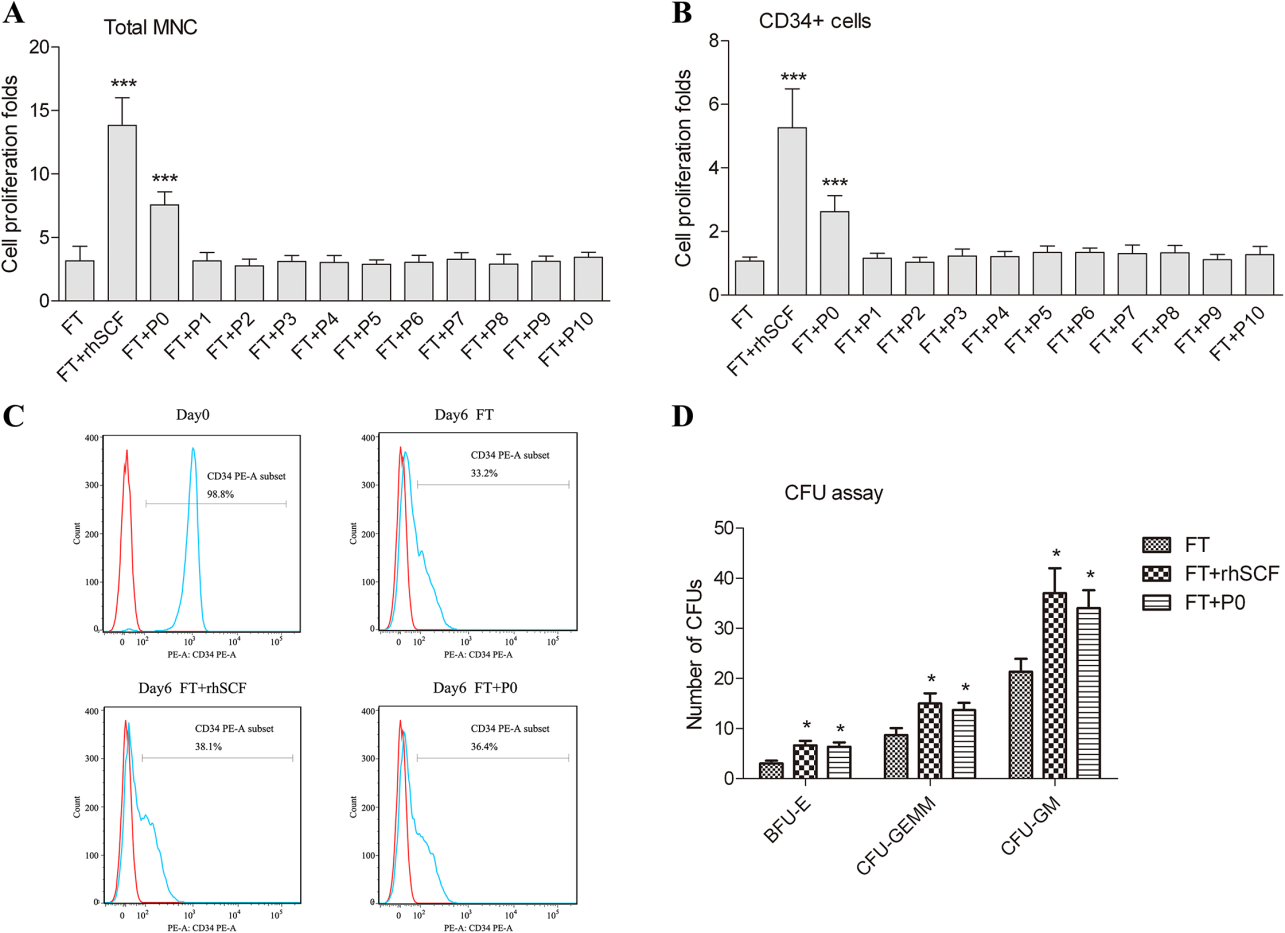
The effects of the synthetic peptides on CD34^+^ cell expansion ex vivo and CFU assay. Affinity-purified umbilical cord blood CD34^+^ cells from four donors were cultured in FT (FL and TPO) basal medium, alone, or supplemented with either rhSCF (100 ng/ml), or a synthetic peptide (100 ng/ml). The numbers of total mononucleated cells (MNC; A) and CD34^+^ cell (B) were determined using flow cytometry on day 6. Cell proliferation fold was calculated as the fold increase [(after expansion)/ (before expansion)] in cell counts. (C) Representative flow cytometry results showing the purity/proportion of CD34^+^ population before/after culturing. (D) Types of CFU colonies formed from CD34^+^ cells of the three groups. Data represent means ± SD, n = 4. ***, P<0.001, *<0.05, compared to FT basal medium group. One-way ANOVA followed by Dunnett’s multiple comparison test.

**Fig 5:**
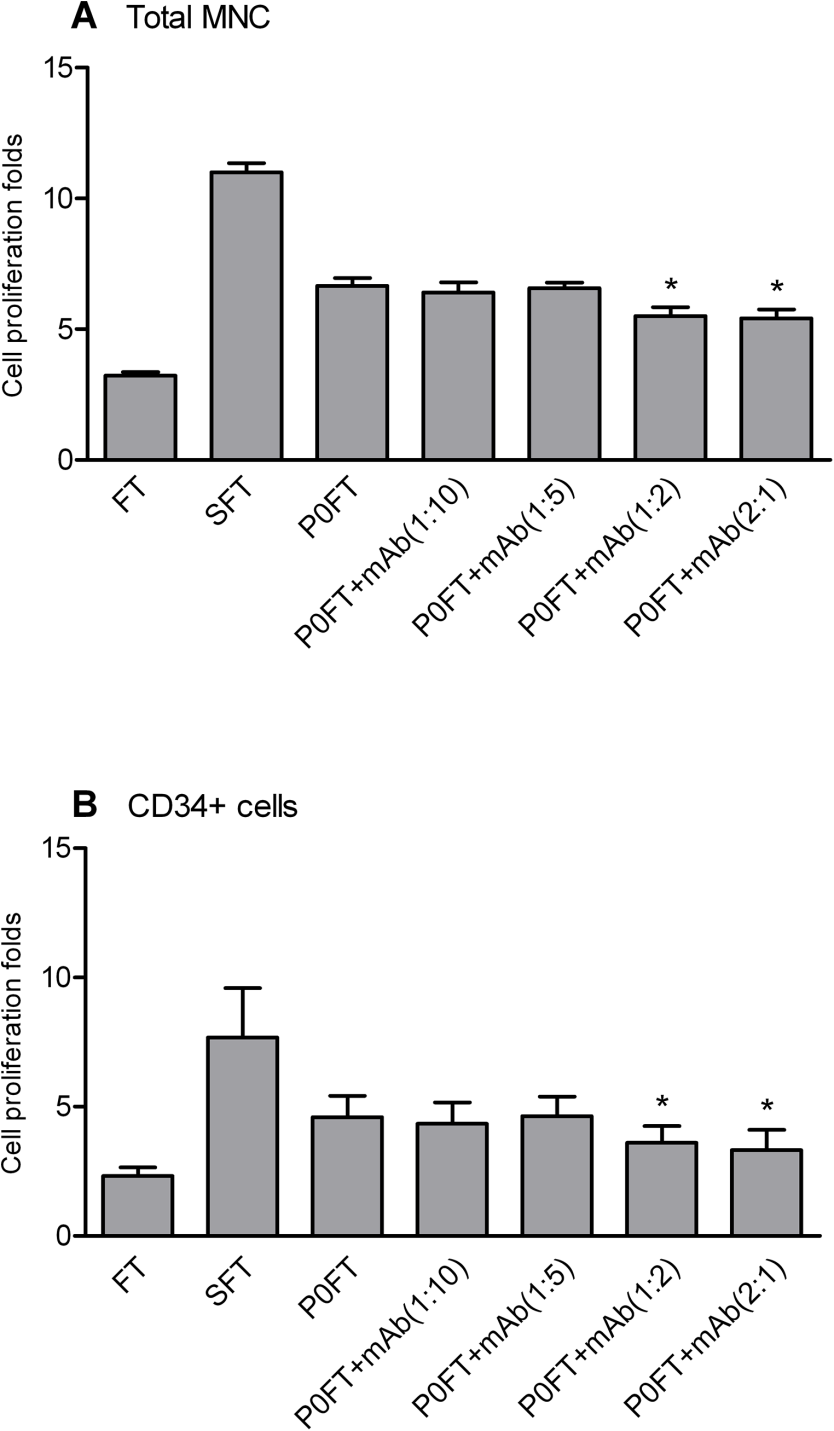
Inhibition of P0’s activity by mAb 23C8. UCB derived CD34^+^ enriched cells were cultured with FT basal medium, with additional supplements of rhSCF (100ng/ml, marked as SFT) or P0 (100ng/ml, marked as P0FT), Anti-rhSCF monoclonal antibody strain 23C8 was also included in the treatment, at 0.1, 0.2, 0.5 and 2 molar fold to P0. The number of total MNC (A) and CD34^+^ cell (B) were calculated on day 6. Data represent means ± SD, n = 3. * P<0.05, mAb treatment groups compared to P0FT group. One-way ANOVA followed by Dunnett’s multiple comparison test.

The ex vivo CD34^+^ cell expansion assay was conducted using affinity-purified UCB CD34^+^ cells from individual donors, with use of rhSCF as the positive control. Total mononucleated cell number and CD34^+^ cell number were counted on day 6. The addition of rhSCF to the basal medium (containing FL and TPO) led to a 4-fold increase in expansion index for total mononucleated cells and a 5-fold increase for CD34^+^ cells, relative to groups with the basal medium alone (Fig 4A and 4B). Of all 11 peptides tested, only P0 was able to stimulate cell expansion. Addition of P0 to the basal medium increased expansion index for both total cells [from 3.15±0.99 to 7.58±0.87 (P<0.001)] and CD34^+^ cells [from 1.07±0.11 to 2.63±0.43 (P<0.001)]. Representative flow cytometry results showed the purity of the CD34^+^ population before and after culturing (Fig 4C). High-purity CD34^+^ cells were used as initial cells for experiments (Fig 4C, day0). After 6 days of culturing ex vivo, CD34^+^ proportion decreased to 30%-40% in all three groups (Fig 4C). Results of the CFU Assay (Fig 4D) showed that the cells from both FT+rhSCF group and FT+P0 group could form various colonies, including BFU-E, CFU-GEMM and CFU-GM. Both groups produced significantly greater numbers of CFU colonies than the FT group (negative control) did; but there was no significant difference between the two groups, indicating that P0 is similar to rhSCF in its ability to stimulate human CD34^+^ cell expansion and to maintain the multi-lineage differentiation potential. Furthermore, the stimulatory activity of P0 toward CD34^+^ cells, like that of the full length SCF, was inhibited by the mAb 23C8 at molar ratio of more than 0.5 molar fold over P0 (Fig 5).

### Ability of P0 to compete against rhSCF in the ex vivo expansion assay

**Fig 6:**
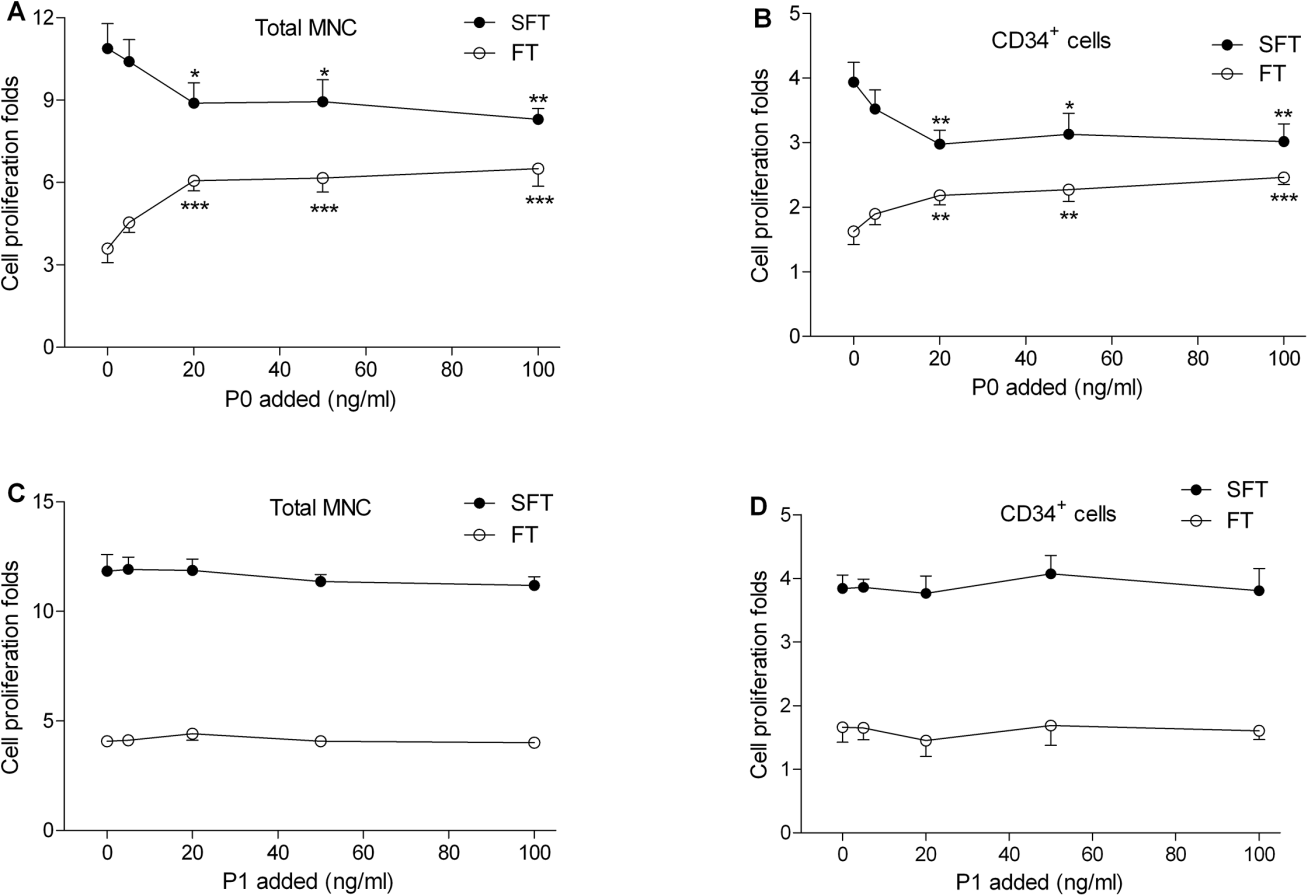
Inhibition of rhSCF activity by P0. Affinity-purified umbilical cord blood CD34^+^ cells from three donors were cultured in FT basal medium alone (FT arm), or supplemented with 100 ng/ml rhSCF (SFT arm), and with varying amounts of P0 (0–100 ng/ml) (A, B) or P1 (0–100 ng/ml) (C, D). The numbers of total MNC (A, C) and CD34^+^ cell (B, D) were determined on day 6. Data represent means ± SD, n = 3. *, **, ***, P<0.05, P<0.01, P<0.001, respectively, compared to corresponding control group (no P0 or P1 added). One-way ANOVA followed by Dunnett’s multiple comparison test.

The results in Fig 4 suggested that, although P0 is capable of stimulating CD34^+^ cell expansion, it is less potent than the full length rhSCF. Thus, we reasoned that, if P0 and rhSCF stimulate CD34^+^ cell expansion by binding to the same molecular target, presumably the SCF receptor KIT, then the presence of P0 may block the ability of rhSCF to stimulate cell expansion. As shown in Fig 6A and 6B, while addition of P0 to the basal medium at 20–100 ng/ml led to further stimulation of cell expansion, addition of P0 to basal medium that already contains rhSCF led to partial, but significant inhibition of cell expansion. However, P1 (as a negative control), showed no effect on cell expansion (Fig 6C and 6D).

## Discussion

Human SCF has two major isoforms, one soluble and one transmembrane; they are generated by alternative splicing, which either retains or excludes a proteolytic cleavage site between the extracellular domain and the transmembrane domain that allows the production of the shorter, soluble isoform [14, 27). SCF binds to and activates KIT, which is a type III transmembrane receptor tyrosine kinase [28, 29), and is expressed in HSCs at all stages of development [22). The rhSCF produced in our laboratory was recombinant soluble human SCF, which consisted of 164 amino acids (Fig 3A). The bioactivity of this rhSCF, which is fully competent in stimulating human CD34^+^ cell expansion ex vivo, could be blocked by the novel SCF monoclonal antibody 23C8 produced in our previous study [24).

Our initial analysis of the putative binding site of 23C8 on rhSCF localized it to the first 104 amino acids from the NH2-terminus [24). Given that the western blot assay used in that earlier study would only detect primary structure, rather than three-dimensional structure, we have performed ELISA in the present study, which is capable of detecting conformational as well as linear epitopes. Furthermore, to narrow the search to a smaller region of rhSCF, we screened 11 overlapping peptides (ranging from 13 to 26 amino acids) that cover the full length of rhSCF. The present result, which identified peptide 0, corresponding to amino acids 39–56 of the rhSCF, as the location of the binding site, is consistent with the earlier finding.

P0 appeared to be less potent than the full-length rhSCF protein both in ELISA and in the ex vivo cell expansion assay. For the screening assays, P0 and full-length rhSCF were added in equal amounts (100 ng/ml), which meant that the molar concentration of P0 was ~10 fold higher than that of rhSCF. However, under those conditions, rhSCF was still more robust than P0 in binding to the anti-SCF antibody in ELISA, or to their cellular target (presumably KIT) in the ex vivo expansion assay. It is likely that, although none of the other peptides analyzed had significant, direct interactions with the anti-SCF, they may contribute to the overall conformational structure of the full-length rhSCF protein, in ways that allow stronger interaction of the protein with the antibody than P0 does. Similarly, in the CD34^+^ cell expansion assay (Fig 4), even though P0 was the only peptide that was able to further stimulate cell proliferation, the other regions of the protein may be important in maintaining optimal conformation for activation of the SCF receptor, and the consequent enhancement of cell expansion.

Notably, peptide 0 contained three lysine residues (two at N-terminus and one at C-terminus) that were not present in SCF; these were added to increase the isoelectric point and solubility of the peptide. These added residues are unlikely recognized by the antibody, given that the antibody recognized the full length protein, which does not contain these residues. However, it is possible that these added sequence might also contributed to a reduction in the ability of P0 to activate CD34^+^ cell expansion, or to compete against rhSCF for binding to the anti-SCF antibody.

SCF activates its receptor KIT by inducing KIT dimerization, which leads to conformational changes in the membrane proximal immunoglobulin-like domains [30). The binding sites between SCF and KIT have been identified through analysis of crystal structures of binding complexes between SCF and KIT [30, 31). Based on the crystal structure, SCF homodimer interacts with the D1-D3 region of KIT to induce KIT dimerization. The three binding sites between mouse SCF and KIT involve multiple residues in SCF, including N97-R104 (to KIT D1), I50-L88 (to KIT D2), and N6-N11 and K81/D85 (to KIT D3). The corresponding contact sites in human SCF are contained in multiple peptides that were analyzed here for ability to activate CD34^+^ cell expansion. The fact that the peptide P0 alone, but no other peptide analyzed, including those containing the binding sites to KIT, such as P1 (binds to D3) and P6 (binds to D1), was able to activate KIT-like function in our cell system suggests that it alone can induce receptor dimerization, albeit at lower efficiency than the full length SCF protein. Furthermore, the residues directly contacting D2 (V50/D54) in this region are contained in both P0 (L39-L56) and P4 (V50-N72, yet only P0 was apparently able to bind KIT, which suggested that the sequence before V50 (part of the B-helix) is important for maintaining correct conformation for binding to KIT. In that regard, further demonstration of direct binding of P0 to KIT and structural analysis of the binding complex may guide future attempts to design more effective small molecule ligands for KIT.

## Conclusion

Residues 39–56 of rhSCF comprise a critical functional region for its ability to enhance expansion of human UCB CD34^+^ cells. The peptide P0 is a potential candidate for further development as a synthetic substitute for rhSCF in clinical applications.
